# A test of the mediating effects of social interaction, physical fitness, and alleviation of negative emotions in the influence of physical exercise on residents' subjective wellbeing—empirical analysis based on CGSS 2023

**DOI:** 10.3389/fpsyg.2025.1695137

**Published:** 2025-11-26

**Authors:** Shutong Zhao, Haiyan Huang

**Affiliations:** 1 College of Physical Education, West Anhui University, Lu'an, China; 2 School of Economics and Management, Shanghai University of Sport, Shanghai, China; 3 School of Management, Beijing Sport University, Beijing, China

**Keywords:** subjective wellbeing, physical exercise, physical fitness, sports industry, psychological effects, social interaction, Chinese General Social Survey

## Abstract

**Introduction:**

Participation in physical exercise (PE) can effectively enhance residents' subjective wellbeing; however, additional research is warranted to elucidate the mechanisms by which exercise participation influences population wellbeing. This study aims to investigate the impact of physical exercise on residents' subjective wellbeing and explore its mediating mechanisms, offering deeper insights into the psychological effects of exercise. Additionally, it provides valuable guidance for enhancing public welfare and promoting social harmony.

**Methods:**

This cross-sectional, observational study analyzed the nationally representative 2023 China General Social Survey (CGSS). In line with the CGSS 2023 sampling frame, “residents” were defined as adults aged ≥18 years living in private households across mainland China in both urban and rural areas. After sample screening, we retained 5,377 valid observations. To examine the relationship between physical exercise and subjective wellbeing and its underlying mechanisms, we employed multiple linear regression, ordered probit models, propensity score matching (PSM), stepwise regression, and the Karlson–Holm–Breen (KHB) decomposition.

**Results:**

The findings indicated that physical exercise was positively associated with Chinese adults' subjective wellbeing. The strength of this association differed across subgroups: individuals with a high school education or less, low income, men, and middle-aged adults showed larger positive associations with subjective wellbeing. Furthermore, the overall association was partially accounted for by differences in social interaction, physical fitness, and negative emotions; these factors operated jointly in relation to subjective wellbeing.

**Conclusions:**

This study identified a positive association between physical exercise and Chinese adults' subjective wellbeing, with social interaction, physical health, and the alleviation of negative emotions functioning as mediating mechanisms in this association. Accordingly, it is recommended that governments, schools, and all sectors of society expand and diversify physical activity (PA) programs and cultivate supportive environments that promote regular exercise, thereby improving population health and wellbeing.

## 1 Introduction

Subjective wellbeing (SWB) refers to the degree of satisfaction and pleasure individuals experience when evaluating their quality of life and perceived value of life. Beyond its established links to mental and physical health, it also serves as a key indicator of national welfare (Skevington and Böhnke, 2018). In China, enhancing public wellbeing and fulfilling aspirations for a better life have long been policy priorities. However, despite sustained economic growth and rising gross domestic product (GDP), improvements in citizens' wellbeing have not kept pace and, on some metrics, have even declined. The United Nations World Happiness Report ranked China 60th among 143 countries and regions in 2024, reflecting a notable reversal that nearly offsets prior gains. Therefore, against the transition to high-quality economic development, strengthening public wellbeing has become an urgent and central task for China's socioeconomic governance.

Physical activity (PA) is the broadest construct, referring to any bodily movement produced by skeletal muscles that expends energy across the leisure, transport, occupational, and domestic domains. Physical exercise (PE) is a purposive subset of PA—planned, structured, and repetitive—undertaken to improve or maintain physical fitness (Caspersen et al., 1985). This study focuses on PE, defined as residents' planned, structured, and repetitive activities aimed at enhancing physical fitness. The term “residents” refers to adults aged 18 years or older living in mainland China. In exercise psychology, the mental health implications of PE have long been central, and substantial literature indicates that PE helps maintain and improve individual health (Li et al., 2021; Herbert, 2022) and provides an important context for building social networks. Extensive evidence also links PE to subjective wellbeing; regular participation is associated with better mental health, fewer depressive symptoms, and higher psychological wellbeing (Guo et al., 2024; Zhou et al., 2022; Yang et al., 2023). However, some studies report null or even negative associations in certain populations (Wicker and Frick, 2015). Collectively, these findings suggest that the PE–SWB relationship is complex, context-dependent, and likely moderated or attenuated by other psychological factors.

Against the backdrop of China's extensive promotion of the Healthy China and National Fitness initiatives, investigation of the effects of PE on Chinese adults' SWB and the underlying mechanisms is of considerable significance. This study investigates the effects of PE on SWB and the underlying mechanisms. The marginal contributions of this study are reflected in the following three dimensions. First, it enriches research on Chinese adults' SWB within the Healthy China framework from a macro perspective and extends the inquiry into factors influencing SWB. Second, it identifies the mediating pathways—social interaction, physical fitness, and the alleviation of negative emotions—through which PE influences SWB, thereby clarifying the mechanisms of influence. Third, it reveals heterogeneous effects across groups with different structural characteristics, providing more granular evidence for policymaking and addressing the shortcomings of existing studies in guiding policy practice.

### 1.1 Subjective wellbeing

Wellbeing is a complex psychological construct with hierarchical and structural characteristics (Vázquez et al., 2009), representing an individual's subjective experience that encompasses multiple dimensions, such as life satisfaction and emotional states (Veenhoven, 2011). It has long been a focal topic in disciplines such as psychology and sociology. Easterlin first introduced the concept of SWB from psychology into economics, treating it as a crucial indicator of welfare. This development has marked the emergence of SWB as a key research topic in economics. In academic research, SWB is commonly used to quantify happiness, and policy evaluations are conducted by examining cross-sectional and longitudinal differences in individuals' perceptions of wellbeing (Ryff, 1989; Seaford, 2013). Consequently, an increasing number of researchers have employed quantitative methods to examine the factors influencing SWB.

In the existing literature, the factors influencing SWB are generally explored from micro- and macro-level perspectives. At the micro level, variables such as gender, age, marital status, health, and income are recognized as key determinants of SWB across many populations. Carlson and Hans (2020), using data from Western countries, argued that in dual-earner households, women tend to undertake more domestic work than men, contributing to lower SWB for women. Similarly, Larsson et al. (2019) conducted a Swedish two-year follow-up study and found that individuals experiencing chronic or severe pain reported significantly lower happiness than those in good physical health. Overall, micro-level characteristics, such as health status and gender roles, play a foundational role in shaping individual SWB across different sociocultural contexts. At the macro level, structural factors such as income inequality, social equity, environmental regulation, and government scale have been shown to influence SWB, although the findings vary across countries. Yu and Chen (2016) found that, among the Chinese population, negative emotions were influenced by both relative and absolute income, whereas happiness and life satisfaction were associated only with relative income. Li et al. (2019), using data from the Chinese General Social Survey (CGSS), reported a positive relationship between fiscal transparency and SWB in China. These findings collectively underscore the importance of macro-level socioeconomic and institutional conditions in shaping the broader landscape of SWB, particularly within the Chinese context.

In summary, existing studies on the determinants of SWB have predominantly adopted micro- and macro-level perspectives. Although a few studies have associated PE with SWB, the mechanisms underlying this relationship remain underexplored.

### 1.2 The relationship between physical exercise and subjective wellbeing

A substantial body of research has shown that PE benefits mental health. Trajković et al. (2023) reported that PE enhances healthy life expectancy, quality of life, and cognitive performance in diverse populations. More broadly, PE yields beneficial psychological outcomes, such as higher life satisfaction, more positive affect, improved self-evaluation, stronger self-efficacy and self-confidence, perceived physical competence, and better socioemotional skills among young people. Scholarship has also examined happiness associated with PE during specific periods, particularly under social isolation. Engaging in appropriately dosed exercise during isolation strengthens self-efficacy and self-control (Živković et al., 2022), which helps reduce the symptoms of depression and anxiety. Such activities further promote self-acceptance, facilitating progress toward intrinsic goals and a sense of fulfillment (Jiménez-Pavón et al., 2020). During the pandemic, home-based recreational exercise and small-group activities created opportunities for emotional expression and sharing, encouraged relaxation, and alleviated psychological stress (Ginoux et al., 2021).

Based on the above, we propose the following hypothesis: H1: Physical exercise is positively associated with Chinese adults' subjective wellbeing.

### 1.3 Heterogeneity hypothesis of the impact of physical exercise on subjective wellbeing

SWB is influenced by individual- and societal-level factors. At the individual level, factors include income, age, gender, education, and health (Das et al., 2020), whereas at the societal level, they encompass unemployment rates, income inequality, and political identity. Therefore, the effects of PE on SWB may be heterogeneous.

First, educational attainment shapes SWB primarily through greater health awareness and behavior change, rather than through large direct effects. As residents become more educated, they tend to reflect on and adjust their health-related behaviors (Kong and Qi, 2017). However, the marginal returns to education diminish, and the direct impact of education on SWB is often modest (Lamu and Olsen, 2016). Moreover, the education–SWB association is strongly conditioned by income and other socioeconomic factors; once these are considered, the link further attenuates (Jivraj and Nazroo, 2014). Notably, PE appears to yield comparatively larger SWB gains among individuals with lower educational attainment (Pu et al., 2023).

Second, income is a salient yet contested correlate of SWB. A large body of literature documents a positive income–SWB gradient (Cramm et al., 2010), with especially strong associations in developing or transitional economies (Frey and Stutzer, 2002). In contrast, Easterlin (1974) argues—and subsequent work supports—that increases in income over time do not translate into proportional gains in happiness, known as the “Easterlin Paradox.” Related studies further suggest that higher income may not raise SWB directly but can buffer against negative affect, such as worry (Yu and Chen, 2016; Li et al., 2016). These mixed patterns imply that the SWB benefits of participating in PE—and the pathways through which PE operates—are likely to vary by income level.

Third, gender patterns in SWB are heterogeneous, suggesting that the PE–SWB relationship may differ by gender. Prior studies report positive, negative, and null correlations between being female and SWB (Lamu and Olsen, 2016). Concurrent evidence indicates greater emotional reactivity among women: larger fluctuations in both negative and positive affect under comparable conditions (Cameron, 1975). In China, some studies find higher SWB among women than men, plausibly reflecting stronger social role expectations and pressures on men, whereas other scholars argue that heavier household responsibilities may depress women's SWB (Carlson and Hans, 2020). Therefore, the effects of participating in PE on SWB—and the mechanisms through which these effects operate—may differ by gender.

Fourth, age patterns in SWB are heterogeneous and shape how PE relates to wellbeing across the course of life. Some studies report a U-shaped profile that stabilizes around the age of 40 years (Uppal, 2006), whereas others document monotonic positive (Jivraj and Nazroo, 2014), negative (Lamu and Olsen, 2016; Uppal, 2006), or null associations (Olsson et al., 2014). These discrepancies likely reflect differences in physical health status and life-cycle stage, which influence both preferences for—and expected returns from—PE. Young people tend to choose adventurous or competitive sports (Keane et al., 2020), middle-aged adults often prioritize stress relief (Wang et al., 2023; Xue et al., 2025), and older adults favor mind–body activities (e.g., tai chi, fishing, and square dancing). Consistent with these patterns, existing studies generally find that older adults who participate in PE report significantly higher SWB (Xu et al., 2022; Su and Zhao, 2021).

Based on the above, we propose the following hypothesis:

H2: The effects of participation in physical exercise on subjective wellbeing are heterogeneous across Chinese adults, varying by education, income, gender, and age.

### 1.4 Hypothesis on the mediating mechanism of physical exercise on subjective wellbeing

Social network theory holds that individuals are embedded in networks of diverse interpersonal ties through which emotions, information, and resources flow (Band and Rogers, 2024). Participation in PE—particularly in team sports, group classes, and community-based activities—can expand network size, strengthen tie quality, and foster norms of reciprocity and trust, thereby accumulating social capital (Bian, 2020). These exercise contexts also create regular opportunities for interaction that facilitate the emergence of perceived social support (Band and Rogers, 2024; Bian, 2020). Belongingness theory further suggests that forming and maintaining meaningful connections engender positive affect and psychological fulfillment, which in turn enhance SWB (Allen et al., 2021). Empirical work indicates that regular PE can build self-confidence and interpersonal trust within social interactions, skills that reinforce engagement with others and indirectly broaden one's social networks (Bian, 2020). Beyond these social pathways, PE has direct psychological benefits—including stress reduction, mood improvement, and resilience—whose effects are often amplified by the supportive relationships formed around shared PE (Guo, 2025).

Based on the above, we propose the following hypothesis:

H3: Social interaction mediates the relationship between participation in physical exercise and Chinese adults' subjective wellbeing.

Although research on health and wellbeing has long emphasized mental health correlates, a growing body of work highlights the contribution of physical health to subjective SWB. Regular PE confers well-established health benefits, with particularly pronounced gains among individuals living with chronic conditions such as cardiovascular disease, diabetes, cancer, hypertension, obesity, depression, and osteoporosis (Penedo and Dahn, 2005). Previous studies indicate that PE can enhance SWB by improving physical functioning and psychological wellbeing, whereas insufficient exercise is linked to more frequent negative emotions (Meng et al., 2025). Consistent with this evidence, Penedo and Dahn (2005) reported that individuals who engage in regular PE achieve better outcomes across multiple physical conditions. Likewise, randomized clinical trials of PE interventions show improvements in overall and health-related quality of life, functional capacity, and emotional wellbeing. Taken together, these findings suggest that PE affects SWB not only through mental health pathways but also via tangible gains in physical health.

Based on the above, we propose the following hypothesis:

H4: Physical fitness mediates the relationship between participation in physical exercise and Chinese adults' subjective wellbeing.

Active participation in PE strengthens social connectedness and positive affect, two pathways consistently linked to higher SWB and life satisfaction (Helliwell and Putnam, 2004). In line with the broaden-and-build theory, the positive emotions elicited by PE broaden cognitive and behavioral repertoires and help individuals accumulate psychological and social resources, thereby buffering depressive and discouraging states (Fredrickson and Losada, 2005). A large empirical literature further shows that PE reduces depressive and anxiety symptoms while enhancing resilience through stress relief, mood regulation, and cumulative physical-health gains (Lee et al., 2022). For example, Hassmén et al. (2000) reported that exercising at least two to three times per week is associated with markedly lower depression, anger, cynical mistrust, and stress than exercising less often or not at all. Herbert et al. (2020) found an inverse association between regular PE and self-reported depression, anxiety, and perceived stress, along with a higher quality of life and positive emotions. At the neurobiological level, Voss et al. (2011) showed that regular PE can modify brain structure and function, with increases in dopamine, serotonin, and norepinephrine linked to improved affect. These findings position PE as a theory-grounded, evidence-based means to promote mental health and, by extension, elevate SWB.

Based on the above, we propose the following hypothesis:

H5: The alleviation of negative emotions mediates the relationship between participation in physical exercise and Chinese adults' subjective wellbeing.

Based on the review and analysis of existing literature, this study proposes three hypothetical mechanisms through which PE may influence Chinese adults' SWB: social interaction, physical fitness, and relief from negative emotions. Given that the proposed hypotheses suggest that participation in PE may be positively associated with SWB and that the three variables—social interaction, physical fitness, and relief from negative emotions—may each serve as mediators in the relationship between PE and SWB, it is possible that these variables may collectively play a mediating role.

Based on the above, we propose the following hypothesis: H6: Participation in physical exercise strengthens social interaction, improves physical fitness, and alleviates negative emotions, all of which contribute to Chinese adults' SWB.

**Figure 1:**
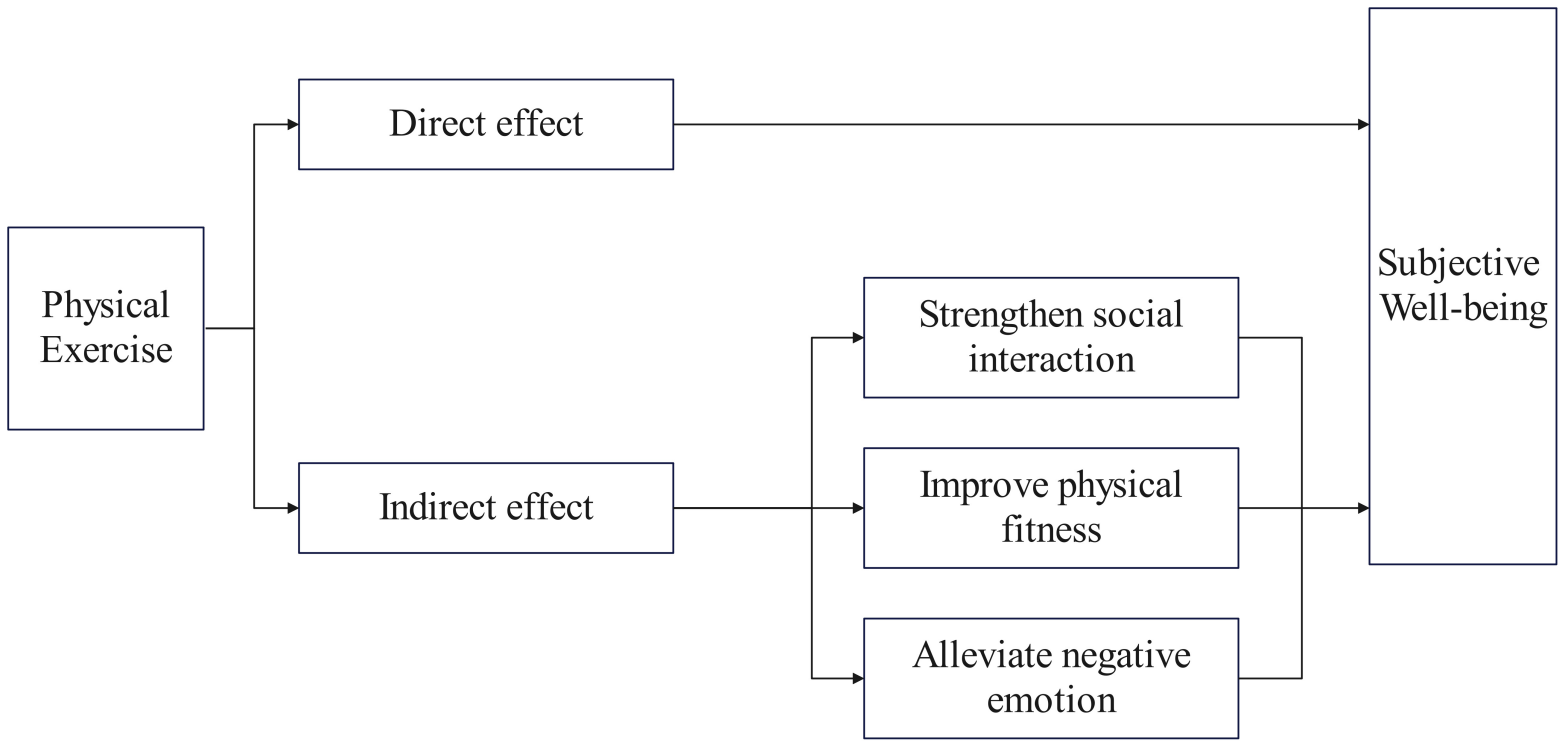
Conceptual model.

Figure 1 presents the theoretical framework linking physical exercise and subjective wellbeing.

## 2 Materials and methods

### 2.1 Sampling

Data for this study were drawn from the Chinese General Social Survey (CGSS), a nationally representative, cross-sectional survey of individuals in mainland China. Administered by the Department of Sociology at the Renmin University of China, the CGSS is publicly available and was launched with a baseline wave in 2003. The survey systematically collected multilevel information—social, community, family, and individual—aimed at documenting patterns of social transformation. Using a multistage, stratified, probability-proportional-to-size (PPS) sampling design, it interviewed approximately 10,000 respondents across 125 counties. As the most recent survey year, the 2023 CGSS data provide a timely and comprehensive picture of the research context, with strong representativeness and analytical value. For this analysis, the 2023 wave of the survey was used, in which 11,326 respondents from 31 provinces completed the questionnaire. After excluding missing responses (e.g., ‘do not know' and ‘refuse to answer') and outliers, the final analytic sample comprised 5,377 cases. Table 1 presents the sample's descriptive characteristics. The data analysis was conducted using Stata 18.0.

**Table 1:** Demographic characteristics of the sample (*N* = 5,377).

**Variables**	**Category**	**Frequency**	**Percentage**
Gender	Male	2,418	44.97
	Female	2,959	55.03
Age	Youth (18–34 years old)	775	14.41
	Middle-aged adults (35–64 years old)	2,866	53.30
	Elderly (≥65 years old)	1,736	32.29
Marital status	Married	3,871	71.99
	Unmarried and other	1,506	28.01
Educational attainment	Primary school and below	1,843	34.28
	Junior high school	1,556	28.94
	Senior high school	997	18.54
	University	923	17.17
	Graduate studies and above	58	1.08
Income level	Low income (annual income ≤ 9,000 yuan)	1,865	34.68
	Middle income (annual income >9,000 and ≤ 80,000 yuan)	2,962	55.09
	High income (annual income > 80,000 yuan)	550	10.23
Household registration	Rural	3,210	59.70
	Urban	2,167	40.30
Region of residence	Eastern region	1,481	27.54
	Central region	1,416	26.33
	Western region	1,829	34.02
	Northeast region	651	12.11
Nation	Han nationality	4,883	90.81
	National minority	494	9.19

### 2.2 Measurements

#### 2.2.1 Dependent variable

This study used subjective wellbeing (SWB) as the dependent variable. SWB was measured using CGSS 2023 item A36: “In general, do you think your life is happy?” Response options were “very unhappy,” “relatively unhappy,” “neither happy nor unhappy,” “relatively happy,” and “very happy.” These categories were coded as 1–5, respectively. Higher scores indicate greater SWB.

#### 2.2.2 Independent variable

Physical exercise (PE) served as the independent variable. The variable was measured using item A3009 from the 2023 Chinese General Social Survey (CGSS), which asks, “In the past year, have you frequently participated in physical exercise during your leisure time?” The response options ranged from 1 to 5: 1 = “Never,” 2 = “Several times a year or less,” 3 = “Several times a month,” 4 = “Several times a week,” and 5 = “Every day.”

#### 2.2.3 Mediating variable

Social interaction served as the mediating variable. The variable was measured using item A3007 from the 2023 Chinese General Social Survey (CGSS), which asks, “In the past year, have you frequently convened with friends during your leisure time?” The response options ranged from 1 to 5: 1 = “Never,” 2 = “Several times a year or less,” 3 = “Several times a month,” 4 = “Several times a week,” and 5 = “Every day.” Higher scores reflect a greater degree of social interaction.

Physical fitness served as the mediating variable. Physical fitness was mainly measured using self-reported health scores. In the CGSS, physical fitness was assessed using item A15, “How would you rate your current physical health?” The response options ranged from 1 to 5, corresponding to the categories “Very unhealthy,” “Relatively unhealthy,” “Fair,” “Relatively healthy,” and “Very healthy.” Higher scores reflect better physical fitness.

The alleviation of negative emotions served as the mediating variable. This variable was measured using item A17: “How often have you felt depressed or down in the past four weeks?” The response options ranged from 1 to 5, corresponding to the categories “Always,” “Often,” “Sometimes,” “Rarely,” and “Never.” Lower scores indicated more severe negative emotions among the participants.

#### 2.2.4 Control variables

Several control variables were selected based on previous studies (Zhao et al., 2022; Li et al., 2023). These variables included gender, age, marital status, educational attainment, annual personal income, household registration, and engagement in sports viewership.

### 2.3 Data analysis

#### 2.3.1 Ordinary least-squares (OLS) model

This study constructed the following baseline regression model:


$$\begin{array}{l} SWB_{i}=a_{1}+a_{2}PE_{i}+a_{3}C_{i}+\varepsilon_{i} \end{array}$$ (1)

Where *SWB*_*i*_ denotes the subjective wellbeing of the *i*-th resident; *PE*_*i*_ represents the physical exercise participation of the *i*-th resident; *C*_*i*_ denotes the control variables; *a*_1_, *a*_2_ and *a*_3_ represent the parameters to be estimated; and ε_*i*_ denotes the random disturbance term.

#### 2.3.2 Ordered probit model

We constructed the following Ordered Probit model:


$$\begin{array}{l} SWB_{i}*=\beta_{1}+\beta_{2}PE_{i}+\beta_{3}C_{i}+\varepsilon_{i} \end{array}$$ (2)

where *SWB*_i_* denotes the latent variable representing the *i*-th resident's subjective wellbeing; β_1_,β_2_,β_3_ are the parameters to be estimated; and ε_*i*_ represents the random disturbance term. Because the *SWB* is categorized into five levels, four thresholds *v*_1_,*v*_2_,*v*_3_,*v*_4_ can be set. The relationship between the latent variables *SWB*_i_* and *SWB*_*i*_ is expressed as follows:

When ${SWB}_{i}^{*}\leq v_{1}$, the corresponding *SWB* level is “very unhappy.”

When $v_{1}<SWB_{i}^{*}\leq v_{2}$, the corresponding *SWB* level is “relatively unhappy.”

When $v_{2}<SWB_{i}^{*}\leq v_{3}$, the corresponding *SWB* level is “neither happy nor unhappy.”

When $v_{3}<SWB_{i}^{*}\leq v_{4}$, the corresponding *SWB* level is “relatively happy.”

When $SWB_{i}^{*}>v_{4}$, the corresponding *SWB* level is “very happy.”


$$SWB_{i}=\{\begin{array}{c} 1,SWB_{i}^{*}\leq v_{1}\\ 2,v_{1}<SWB_{i}^{*}\leq v_{2}\\ 3,v_{2}<SWB_{i}^{*}\leq v_{3}\\ 4,v_{3}<SWB_{i}^{*}\leq v_{4}\\ 5,v_{4}<SWB_{i}^{*} \end{array}$$ (3)

Assuming that the random disturbance term follows a standard normal distribution, and *x* represents all explanatory variables, the probability of Chinese adults' SWB at each level can be expressed as follows:


$$\{\begin{array}{c} P(SWB_{i}=1|x)=\varphi (v_{1}-x\beta )\\ P(SWB_{i}=2|x)=\varphi (v_{2}-x\beta )-\varphi (v_{1}-x\beta )\\ P(SWB_{i}=3|x)=\varphi (v_{3}-x\beta )-\varphi (v_{2}-x\beta )\\ P(SWB_{i}=4|x)=\varphi (v_{4}-x\beta )-\varphi (v_{3}-x\beta )\\ P(SWB_{i}=5|x)=1-\varphi (v_{4}-x\beta ) \end{array}$$ (4)

#### 2.3.3 Mediation effect model

We constructed the following mediation effect model:


$$\begin{array}{l} M_{i}=b_{i}+b_{2}PE+b_{3}C_{i}+\varepsilon_{i} \end{array}$$ (5)

$$\begin{array}{l} SWB_{i}=c_{i}+c_{2}PE+c_{3}M_{i}+c_{4}C_{i}+\varepsilon_{i} \end{array}$$ (6)

where *M*_*i*_ represents the mediating variables, including social interaction, physical fitness, and alleviation of negative emotions; *b*_2_×*c*_3_ denotes the mediation effect of physical exercise participation on SWB. The remaining variables retain the same meanings as those defined in Equation 1.

## 3 Results and analysis

### 3.1 Multiple regression analysis

As shown in Table 2, whether SWB is treated as a continuous variable using ordinary least squares (OLS) estimation (Column 1) or as an ordered categorical variable using an ordered Probit model (Column 3), the coefficient for PE remains positive and statistically significant at the 1% level. After including the control variables in the models (Columns 2 and 4), the coefficient for PE continues to be positive and statistically significant at the 1% level. These results indicate a significant positive association between PE and SWB among Chinese adults, which is consistent with H1.

**Table 2:** Multiple regression results.

**Variables**	**SWB**			
	**OLS model**		**Ordered Probit model**	
	**(1)**	**(2)**	**(3)**	**(4)**
PE	0.074^***^ (0.000)	0.057^***^ (0.000)	0.095^***^ (0.000)	0.076^***^ (0.000)
Gender		−0.043^*^ (0.067)		−0.053^*^ (0.085)
Age		0.078^***^ (0.000)		0.120^***^ (0.000)
Marital status		0.103^***^ (0.000)		0.128^***^ (0.0002)
Educational attainment		0.080^***^ (0.000)		0.097^***^ (0.000)
Annual personal income		0.064^***^ (0.001)		0.068^***^ (0.010)
Household registration		0.043 (0.109)		0.060^*^ (0.098)
Watching sports competitions		0.037^**^ (0.030)		0.056^**^ (0.021)
Constant	3.713^***^ (0.000)	3.167^***^ (0.000)		
*N*	5,377			

SWB, Subjective wellbeing; PE, Physical Exercise. *p*-values in parentheses; ^*^p < 0.1, ^**^p < 0.05, ^***^p < 0.01.

### 3.2 Heterogeneity test

#### 3.2.1 Heterogeneity test in educational attainment

As shown in Table 3, participation in PE significantly impacts the SWB of Chinese adults with different educational levels. Specifically, Chinese adults with primary school or below, junior high school, senior high school, and university education exhibit statistically significant effects of PE on their SWB, all at the 1% significance level. The effects, ordered from lowest to highest magnitude, are as follows: university (0.046) < primary school or below (0.054) < junior high school (0.053) < senior high school (0.074). However, PE participation does not significantly influence the SWB of Chinese adults with a graduate education or higher. This finding highlights the heterogeneous effects of PE on SWB across educational levels.

**Table 3:** Heterogeneity test results of educational attainment.

**Variables**	**SWB**				
	**Primary school and below**	**Junior high school**	**Senior high school**	**University**	**Graduate studies and above**
	**(1)**	**(2)**	**(3)**	**(4)**	**(5)**
PE	0.054^***^ (0.000)	0.053^***^ (0.000)	0.074^***^ (0.000)	0.046^***^ (0.009)	−0.021 (0.830)
Constant	2.665^***^ (0.000)	3.339^***^ (0.000)	3.506^***^ (0.000)	3.735^***^ (0.000)	3.984^***^ (0.000)
**Control covariates**	**Yes**				
*N*	1,843	1,556	997	923	58
adj. *R*^2^	0.033	0.019	0.030	0.014	−0.076

SWB, Subjective wellbeing; PE, Physical Exercise. *p*-values in parentheses; ^***^p < 0.01.

#### 3.2.2 Heterogeneity test in income levels

In this study, respondents' annual personal income was used to divide the sample into three income strata based on the 25th percentile (9,000 yuan) and the 75th percentile (80,000 yuan): Chinese adults with an annual income below 9,000 yuan were classified as the “low-income group.” Individuals with incomes between 9,000 yuan and 80,000 yuan were classified as the “middle-income group.” Individuals earning more than 80,000 yuan were classified as the “high-income group.” As shown in Table 4, PE significantly affects the SWB of Chinese adults in both the low- and middle-income groups, with statistically significant effects at the 1% level. The regression coefficients show that the coefficient for PE is larger in the low-income group (0.081) than in the middle-income group (0.046). However, PE does not significantly affect the SWB of adults in the high-income group. Therefore, this study highlights the heterogeneous effects of PE on SWB across income levels.

**Table 4:** Heterogeneity test results of income level.

**Variables**	**SWB**		
	**Low-income**	**Middle-income**	**High-income**
	**(1)**	**(2)**	**(3)**
PE	0.081^***^ (0.000)	0.046^***^ (0.000)	0.017 (0.443)
Constant	3.022^***^ (0.000)	3.325^***^ (0.000)	3.730^***^ (0.000)
**Control covariates**	**Yes**		
*N*	1,865	2,962	550
adj. *R*^2^	0.031	0.026	0.006

SWB, Subjective wellbeing; PE, Physical Exercise. *p*-values in parentheses; ^***^p < 0.01.

#### 3.2.3 Heterogeneity test in gender

Table 5 shows that the SWB effects of PE are significantly higher for males than for females. Sex differences in emotion regulation may arise from biological factors (Christiansen et al., 2022). It has been shown that men and women exhibit differences in neurotransmitter levels, hormone levels, and brain structure and function. These differences may significantly affect emotional regulation and stress-coping mechanisms. Men are more likely to enhance physical strength and competitive abilities through exercise, whereas women may focus more on health maintenance and social interaction (Feraco et al., 2024). These differences may also contribute to the variation in SWB effects.

**Table 5:** Heterogeneity test results of gender.

**Variables**	**SWB**	
	**Male**	**Female**
	**(1)**	**(2)**
PE	0.076^***^ (0.000)	0.041^***^ (0.000)
Constant	3.050^***^ (0.000)	3.235^***^ (0.000)
**Control covariates**	**Yes**	
*N*	2,418	2,959
adj. *R*^2^	0.043	0.028

SWB, Subjective wellbeing; PE, Physical Exercise. *p*-values in parentheses; ^***^p < 0.01.

#### 3.2.4 Heterogeneity test in age

The observations were categorized into age groups based on China's classification standards: Youth, 18–34 years; middle-aged adults, 35–64 years; and elderly, ≥65 years. As shown in Table 6, middle-aged individuals experience higher levels of SWB from PE than both the elderly and youth, with elderly adults showing higher levels than youth. Although elderly individuals also derive SWB from PE, their benefits may be relatively weaker because of the declines in physical function and exercise habits. Youths may prioritize academic pursuits, career development, and social interactions, and their SWB gains from PE may be less pronounced because of time constraints and differing life priorities. These results of the heterogeneity test support Hypothesis H2.

**Table 6:** Heterogeneity test results of age.

**Variables**	**SWB**		
	**Youth**	**Middle-aged adults**	**Elderly**
	**(1)**	**(2)**	**(3)**
PE	0.046^**^ (0.044)	0.060^***^ (0.000)	0.053^***^ (0.000)
Constant	3.558^***^ (0.000)	3.050^***^ (0.000)	3.502^***^ (0.000)
**Control covariates**	**Yes**		
*N*	775	2,866	1,736
adj. *R*^2^	0.022	0.062	0.022

SWB, Subjective wellbeing; PE, Physical Exercise. *p*-values in parentheses; ^**^p < 0.05, ^***^p < 0.01.

### 3.3 Robustness test

We conducted the following robustness tests to validate the regression results (Table 7):

First, we modified the regression model and re-evaluated the impact of PE on SWB using an Ordered Logit Model (Columns 1 and 2).
Second, the dependent variable and estimation method were modified. Responses regarding Chinese adults' SWB were recoded into a binary variable, with “relatively happy” and “very happy” assigned a value of 1, and all other responses assigned a value of 0. Logit and Probit models were employed for the regression analysis (Columns 3–6).
Third, additional control variables were included. Four items from the CGSS—“Region of residence,” “Nation,” “Health insurance participation,” and “Social confidence”—were added as control variables for the OLS test (Column 7).

**Table 7:** Robustness test results.

**Variables**	**SWB**						
	**Ordered logit model**		**Logit model**		**Probit model**		**Add control variables**
	**(1)**	**(2)**	**(3)**	**(4)**	**(5)**	**(6)**	**(7)**
PE	0.163^***^ (0.000)	0.132^***^ (0.000)	0.172^***^ (0.000)	0.112^***^ (0.000)	0.098^***^ (0.000)	0.065^***^ (0.000)	0.055^***^ (0.000)
Constant			0.819^***^ (0.000)	−0.822^***^ (0.001)	0.514^***^ (0.000)	−0.429^***^ (0.003)	2.735^***^ (0.000)
Control covariates	No	Yes	No	Yes	No	Yes	Yes
*N*	5,377						5,317

SWB, Subjective wellbeing; PE, Physical Exercise. *p*-values in parentheses; ^***^p < 0.01.

As shown in Table 7, the coefficient of the effect of PE on SWB remains significantly positive.

### 3.4 Endogeneity test

To control for potential confounding variables that may influence the regression results, this study employed Propensity Score Matching (PSM) to reassess the positive effect of PE on SWB. Four matching methods—nearest neighbor matching (*k* = 1), nearest neighbor matching with a caliper (0.05), radius matching (0.05), and kernel matching—were employed to match the treated group (PE participation) and the control group (non-participation in PE).

As shown in Table 8, the results obtained using the four matching methods are consistent, with ATT values of 0.146, 0.146, 0.167, and 0.165, which are all statistically significant at the 1% level. The results from the PSM model not only validate the findings from the multiple regression analysis but also examine the causal relationship between SWB and PE. Therefore, Hypothesis H1 is further supported. This finding further demonstrates that PE has positive psychological effects and broadens the understanding of these effects.

**Table 8:** PSM results.

**Methods**	**Treated (1)**	**Controls (2)**	**ATT/difference (3) = (1)–(2)**	**S.E**.	***t***
Nearest neighbor (*k =* 1)	3.999	3.853	0.146	0.050	2.90^***^
Nearest neighbor matching with caliper (0.05)	3.999	3.853	0.146	0.050	2.90^***^
Radius matching (0.05)	3.999	3.831	0.167	0.040	4.19^***^
Kernel matching	3.999	3.834	0.165	0.043	3.80^***^

The variable for participation in physical exercise has been recoded here, converting it from a five-point variable to a two-point variable. Because of space limitations, the balance sheet for testing matched data is not included in the article. ^***^p < 0.01.

### 3.5 Mediation model results

#### 3.5.1 Mediation effect analysis of a single variable

A stepwise regression method was used to examine the mediating effects. Table 9 presents the results of the mediating effect tests, while Table 10 summarizes the effect sizes of the mediation.

**Table 9:** The mediating effect of PE on Chinese adults' SWB.

**Variables**	**SWB**	**Social interaction**	**SWB**	**Physical fitness**	**SWB**	**Alleviate negative emotion**	**SWB**
	**(1)**	**(2)**	**(3)**	**(4)**	**(5)**	**(6)**	**(7)**
PE	0.055^***^ (0.000)	0.076^***^ (0.000)	0.050^***^ (0.000)	0.078^***^ (0.000)	0.042^***^ (0.000)	0.074^***^ (0.000)	0.041^***^ (0.000)
Social interaction			0.0666^***^ (0.000)				
Physical fitness					0.1668^***^ (0.000)		
Alleviate negative emotion							0.199^***^ (0.000)
Constant	3.245^***^ (0.000)	1.846^***^ (0.000)	3.122^***^ (0.000)	3.353^***^ (0.000)	2.686^***^ (0.000)	2.968^***^ (0.000)	2.655^***^ (0.000)
**Control covariates**	**Yes**						
*N*	5,377						
adj. *R*^2^	0.034	0.167	0.039	0.156	0.076	0.065	0.099

SWB, Subjective wellbeing; PE, Physical Exercise. *p*-values in parentheses; ^***^p < 0.01.

**Table 10:** Summary of mediation effect results.

**Influence path**	**Conclusion**	**Total effect (*a*_2_)**	**Mediating effect (*b*_2_×*c*_3_)**	**Direct effect (*c*_2_)**	**Calculation Equation**	**Proportion of mediating effect (%)**
PE → Social interaction → SWB	a partial mediating effect	0.055	0.005	0.050	(*b*_2_×*c*_3_)/*a*_2_	9.12
PE → Physical fitness → SWB			0.013	0.042		23.33
PE → Alleviating negative emotion → SWB			0.015	0.041		26.76

For the meaning of symbols, see Equations 1, 5, and 6.

SWB, Subjective wellbeing; PE, Physical Exercise.

Columns 2 and 3 of Table 11 display the results of the mediating effect tests for social interactions. Participation in PE shows a significantly positive effect at the 1% level, and social interaction also shows a significant positive effect at the 1% level. This finding indicates that social interaction has a significant mediating effect, specifically a partial one. Therefore, Hypothesis H3 is validated. The direct effect is 0.050, while the mediating effect of PE through enhanced social interaction on SWB is 0.005, representing 9.12% of the total effect.

**Table 11:** Bootstrap test.

**Mediating variables**	**Effect types**	**Observed coefficient**	**Bootstrap std. err**.	**Bias-corrected 95% CI**	
				**Lower**	**Upper**
Social interaction	Indirect effect	0.005	0.001	0.003	0.008
	Direct effect	0.052	0.008	0.036	0.066
	Total effect	0.057	0.008	0.041	0.071
Physical fitness	Indirect effect	0.013	0.002	0.010	0.017
	Direct effect	0.044	0.008	0.029	0.060
	Total effect	0.057	0.008	0.042	0.073
Alleviate negative emotions	Indirect effect	0.014	0.002	0.010	0.019
	Direct effect	0.043	0.007	0.029	0.057
	Total effect	0.057	0.008	0.042	0.073

Columns 4 and 5 of Table 11 display the results of the mediating effect tests for physical fitness. Participation in PE shows a significantly positive effect at the 1% level, and physical fitness also shows a significant positive effect at the 1% level. This indicates that physical fitness has a significant mediating effect, specifically a partial one. Therefore, Hypothesis H4 is validated. The direct effect is 0.042, while the mediating effect of participation in PE through improved physical fitness on SWB is 0.013, representing 23.33% of the total effect.

Columns 6 and 7 of Table 11 display the results of the mediating effect tests for alleviating negative emotions. Participation in PE shows a significantly positive effect at the 1% level, and alleviating negative emotions also shows a significant positive effect at the 1% level. This indicates that alleviating negative emotions has a significant mediating effect, specifically a partial one. Therefore, Hypothesis H5 is validated. The direct effect is 0.041, while the mediating effect of participation in PE through alleviating negative emotions on SWB is 0.015, representing 26.76% of the total effect.

Additionally, the mediating effects were tested using the Bootstrap method. The results of the Bootstrap estimation with 1,000 resamples (Table 11) indicate that the indirect effects of social interaction, physical fitness, and alleviating negative emotions are 0.005, 0.013, and 0.014, respectively. Furthermore, the bias-corrected 95% confidence intervals (CIs) for all indirect effects do not include 0, providing further evidence for the mediating effects of these three variables.

#### 3.5.2 Tests for co-mediation effects—KHB test method

In this study, the KHB method proposed by Karlson, Holm, and Breen was further applied to examine the mediating effects of three variables. The KHB method not only estimates the mediating effect of a single mediator but also evaluates the combined mediating effects of multiple mediators and their respective contributions. As shown in Table 12, the combined mediating effect of social interaction, physical fitness, and the alleviation of negative emotions on SWB through PE is 0.023 (*p* < 0.01), accounting for 39.58% of the total effect. Compared to the mediating effects of individual variables, the combined mediating effects are stronger, and their proportion of the total effect is also greater. Consequently, Hypothesis H6 is supported, which posits that PE influences Chinese adults' SWB by strengthening social interaction, improving physical fitness, and alleviating negative emotions.

**Table 12:** KHB mediating effect test.

**Mediating variables**	**Social interaction, physical fitness, and alleviating negative emotion**
Total effect	0.057^***^ (0.007)
Direct effect	0.035^***^ (0.007)
Indirect effect	0.023^***^ (0.003)
Percentage of mediating effects	Indirect effect/total effect = 39.58%
*N*	5,377

*p*-values in parentheses; ^***^p < 0.01.

## 4 Discussion

This study empirically investigated the impact of PE on SWB and its underlying mechanisms, as well as variations across gender, age, educational background, and income levels. The following is a point-by-point discussion of each research finding.

### 4.1 The impact of physical exercise on subjective wellbeing

The regression results showed that PE had a statistically significant positive effect on Chinese adults' SWB, with a causal relationship between the two variables. These findings aligned with those of previous studies (Trajković et al., 2023; Ginoux et al., 2021; Iwon et al., 2021). This positive effect can be attributed to several psychological and social mechanisms. First, PE enhances individuals' exercise identity and sense of competence, which boosts their self-esteem and wellbeing (Cui, 2025). Second, it improves mental health by reducing depression and strengthening peer relationships, both of which are significant mediators of SWB. Third, PE contributes to a better body image and higher self-esteem, which together create a positive emotional state (Shang et al., 2021). Therefore, the development of mass sports initiatives supports the implementation of the “Healthy China” strategy, improves Chinese adults' social attitudes, and promotes social stability and development.

### 4.2 Heterogeneity in subjective wellbeing

Evidence from our analysis indicated that the wellbeing gains from PE were most pronounced among Chinese adults with a high school education or less. These individuals often face greater economic pressure and are at earlier career stages, making exercise a salient stress-management strategy that substantially improves SWB (Li et al., 2022). In comparison, university-educated adults tend to have stronger economic security and lower day-to-day stress; therefore, the marginal benefit of exercise for wellbeing is smaller. At the graduate level, demanding workloads and tighter schedules often impede regular participation in exercise (Jett et al., 2024), thereby attenuating its effects. These patterns suggest that an education-linked gradient in the wellbeing returns to exercise is shaped by differences in stress exposure, available time, and competing demands.

The association between PE and SWB appears to vary according to income level. For low-income adults, who typically face greater economic pressure and daily challenges, exercise offers an accessible means of stress relief and mood regulation, which produces comparatively larger gains in SWB (Yao and Xiangyu, 2025). In contrast, high-income adults benefit from more favorable economic conditions and lower stress, often obtaining SWB from a wider array of leisure options—such as cultural events and other paid activities—so the marginal return to exercise was smaller (Wheatley and Bickerton, 2024). For middle-income adults, who frequently balance career advancement with family responsibilities, regular exercise helps manage stress and improves affect (Lee et al., 2015), yielding meaningful improvements in SWB. Taken together, these patterns indicate income-contingent mechanisms and highlight the need to tailor exercise promotion strategies according to the constraints and preferences of different socioeconomic groups.

PE had a positive impact on SWB; however, its effects appeared to be more significant in men than in women. This discrepancy may be explained by gendered social and economic roles. Men are often expected to shoulder greater financial responsibilities and face higher levels of occupational competition, making PE a crucial outlet for stress relief and a way to enhance self-confidence. As a result, regular exercise can lead to notable improvements in men's overall sense of wellbeing (Gan and Jiang, 2022). In contrast, women frequently experience dual burdens from both work and family responsibilities. These competing demands may limit their time and energy for PE, whereas societal role expectations and less consistent exercise habits can further reduce the emotional benefits derived from exercise. Consequently, although PE is positively associated with SWB across groups, the estimated magnitude of the association may be comparatively smaller for women than for men.

The positive impact of PE on SWB appeared to be most pronounced among middle-aged adults. While older individuals also benefit from exercise, the effects may be less significant because of age-related declines in physical function and less consistent exercise habits (Yamashita et al., 2019). In contrast, younger people often prioritize academic achievements, career development, and social relationships, which may limit the time and attention they devote to PE. Consequently, the contribution of exercise to their wellbeing may be relatively lower (Tao et al., 2022). Middle-aged adults, who typically face considerable work pressure and family responsibilities, experience higher levels of overall life stress (Lachman et al., 1994). PE serves as an effective coping mechanism, helps alleviate stress, improves mood, and enhances both physical and mental health. Furthermore, individuals in this age group often possess relatively good physical health and well-established exercise routines (Kim et al., 2016), which enable them to participate in regular PE. This consistency not only supports physical wellbeing but also promotes psychological fulfillment (Li et al., 2025) and is associated with higher overall levels of SWB.

### 4.3 The mechanism of physical exercise affecting subjective wellbeing

This study showed that PE indirectly influenced SWB through the mediating roles of social interaction, physical health, and the alleviation of negative emotions. Furthermore, these three mechanisms operated jointly and were associated with SWB.

This study found that PE was positively associated with Chinese adults' SWB, and that social interaction partially accounted for this association. PE provides structured settings for communication and joint activities, expands social networks (Kim et al., 2021), and reinforces collective cohesion. These interactions help mitigate negative social attitudes and increase perceived social support and access to resources for pursuing personal goals (Li and Zhang, 2025). PE also promotes mental and emotional wellbeing by facilitating emotional exchange, conflict resolution, and stress relief. In team-based contexts, rules and task allocation foster durable relational ties, which in turn support the building and maintenance of social relationships (Silva et al., 2013), enhance belonging and identity, and contribute to social stability. These findings are consistent with those of related studies (Zou and Sun, 2011).

PE influenced Chinese adults' SWB by enhancing their physical fitness. The benefits of PE on physical and mental health are widely documented in previous research (Powell, 2018). Improved health status influences Chinese adults' happiness through multiple pathways: by promoting physical health and fitness, PE reduces pain and the economic burden of disease (Iwon et al., 2021) and thereby increases psychological satisfaction and life satisfaction. Better physical condition also supports higher self-esteem and a more favorable body image (Watt and Konnert, 2020), which, in turn, provides greater opportunities to participate in social activities and economic production and strengthens feelings of social integration and economic security.

This study found that PE influenced Chinese adults' SWB by alleviating negative emotions. This interpretation is consistent with prior research: the “amine hypothesis” and the “endorphin hypothesis” posit that appropriately intense PE increases the release of neurotransmitters (e.g., dopamine and endorphins) into circulation, thereby exerting favorable effects on mood (Hattori et al., 1994). Through both physiological and psychological pathways, PE reduces negative emotions and improves the symptoms of anxiety and depression (Blumenthal et al., 1999). Individuals who exercise regularly report more favorable emotional states than non-exercisers; as emotional wellbeing improves with training, life satisfaction increases (Elavsky et al., 2005; Martín-Albo et al., 2012), leading to higher levels of SWB. Moreover, PE strengthens self-efficacy and self-confidence (Elavsky et al., 2005), enabling individuals to cope more effectively with stressors and daily challenges.

The mechanism through which PE influenced SWB could be comprehensively understood in light of the KHB test findings, which suggested that three distinct pathways collectively contributed to Chinese adults' SWB. Accordingly, a “socio-physiological-psychological” tridimensional system model was proposed to elucidate the impact of PE on SWB. These three pathways—physiological, psychological, and social—did not function in isolation; rather, they interacted in ways that might have led to synergistic effects.

### 4.4 Practical implications

Building on these findings, it is crucial to foster collaboration among governments, communities, schools, and enterprises to further enhance Chinese adults' SWB. First, expand population-level participation in PE. Sustain and strengthen the National Fitness Program, embed PE into daily routines, and incentivize enterprises, public institutions, and communities to offer diverse activities. Upgrade public sports facilities—especially in communities, rural areas, and low-income regions—to ensure equitable access and reduce barriers. Finally, widely communicate the physical and mental health benefits of PE through media, schools, and community initiatives, and provide science-based education on safe and effective exercise. Second, targeted strategies are essential to achieve “stratified empowerment.” Prioritize low-income and less-educated groups by expanding outreach and resources in underserved communities and rural areas, offering tailored low-cost programs (e.g., community fitness classes and clubs), and waiving fees at public facilities. For men and middle-aged adults, design engaging options—such as team and competitive sports—and integrate mental health education with PE to reduce work- and life-related stress. For women, provide accessible options, such as yoga and dance, to boost participation, support physical and mental health, and promote gender equity in PE. Third, PE's mediating pathways should be enhanced. Embed social components—community clubs and team competitions—to foster interaction, belonging, and broader networks. Provide community health screening and feedback to guide personalized exercise plans, and pair PE with mental health education to amplify physical and psychological benefits. Finally, integrate PE into school curricula and extracurricular activities to instill lifelong exercise habits and support students' wellbeing.

### 4.5 Limitations

This study has some limitations. First, the measurement of PE in this study was restricted to a single indicator—frequency of participation. Future research could address this limitation by employing multiple indicators, such as duration, intensity, years of participation, and types of exercise, to provide a more comprehensive assessment of PE and its effects on wellbeing. Second, this approach will offer a deeper understanding of how PE influences happiness. This study used cross-sectional data from the CGSS 2023 survey. While a series of tests were conducted to validate the causal and mediating relationships between PE and SWB, the cross-sectional design inherently limited the ability to establish causality. Therefore, the generalizability and scalability of our findings require further validation. Future research could adopt a longitudinal design or conduct more rigorous experimental studies to more definitively verify the causal relationship between these variables. Third, this study provided preliminary insights into how PE was related to Chinese adults' SWB through three mediating variables. However, the complex interactions among these mediating processes, the differential effects across various population groups, and the long-term evaluation of these effects warrant further investigation. Future studies should employ more refined experimental designs, advanced measurement techniques, and interdisciplinary approaches to address these gaps.

## 5 Conclusions

This study drew on a nationally representative CGSS 2,023 sample (*N* = 5,377) and employed rigorous analytic techniques—including multiple regression models, propensity score matching to mitigate selection bias, and advanced mediation analysis with KHB decomposition—to assess whether physical exercise was significantly associated with higher levels of subjective wellbeing among Chinese adults. Importantly, the positive influence of exercise on wellbeing was found to be mediated by strengthened social interaction, enhanced physical fitness, and reduced negative emotions, which together augmented individuals' subjective wellbeing. The identification and quantification of these three concurrent mediating pathways within a single integrated framework is a novel contribution of this study, shedding new light on the multifaceted social, physical, and psychological processes through which exercise may be related to wellbeing. The analysis also indicated heterogeneity in associations across demographic groups; men, middle-aged adults, lower-income individuals, and those with a high school education or below exhibited larger estimated PE–SWB associations. These findings have important implications for public health policies. Specifically, this evidence suggested that promoting physical exercise could elevate life satisfaction among Chinese adults and that national initiatives such as Healthy China should prioritize the groups with the greatest potential gains while leveraging the identified social, physical, and emotional pathways to maximize the population-wide wellbeing benefits of exercise.

## Data Availability

Publicly available datasets were analyzed in this study. This data can be found at: http://cgss.ruc.edu.cn/.
